# Early post-exercise physiological and perceptual responses following different sprint interval training configurations in highly trained national-level swimmers: a randomized crossover study

**DOI:** 10.3389/fphys.2026.1922489

**Published:** 2026-09-09

**Authors:** Lijun Long, Ke Chen, Dongting Jiang, Meiling Tao, Kejian Li, Pei Luo, Yanqing Fu, Hao-Nan Wang

**Affiliations:** 1 Sports Coaching College, Beijing Sport University, Beijing, China; 2 Physical Education Institute, Hunan Normal University, Changsha, Hunan, China; 3 Independent Researcher, Beijing, China; 4 School of Athletic Performance, Shanghai University of Sport, Shanghai, China; 5 School of Coaching, Shanghai University of Sport, Shanghai, China; 6 School of Sport Training, Wuhan Sports University, Wuhan, China; 7 School of Sports Sciences, Beijing Sport University, Beijing, China; 8 School of Education, Beijing Sport University, Beijing, China; 9 Sports Medicine Center, West China Hospital, Sichuan University, Chengdu, China

**Keywords:** blood lactate, heart rate, post-exercise recovery, rating of perceived exertion, sprint interval training, swimming

## Abstract

**Background:**

Sprint interval training (SIT) is widely used in competitive swimming, yet early post-exercise physiological and perceptual responses to different SIT configurations remain incompletely understood. This study compared in highly trained national-level middle- and long-distance swimmers. This study compared early post-exercise rating of perceived exertion (RPE), heart rate (HR), and blood lactate concentration (BLa) responses following four composite SIT configurations in national-level competitive middle- and long-distance freestyle swimmers.

**Methods:**

Fifteen highly trained national-level swimmers participated in this randomized crossover study. Each participant completed four SIT configurations in randomized order, with a 7-day washout period between sessions: 12 × 25 m with 1-min recovery, 10 × 35 m with 80-s recovery, 8 × 50 m with 2-min recovery, and 6 × 75 m with 3-min recovery. The work-to-rest ratio was maintained at 1:4 across configurations. Rating of perceived exertion (RPE), heart rate (HR), and blood lactate concentration (BLa) were measured before exercise and immediately post-exercise and at 1, 3, 5, 7, and 9 min of recovery. Baseline-adjusted linear mixed-effects models were used to examine protocol, time, and protocol × time effects, with post-exercise time treated as a categorical factor.

**Results:**

After adjustment for session-specific baseline values, significant main effects of protocol and time were observed for RPE, HR, and BLa (all p < 0.001). The protocol × time interaction did not reach statistical significance for RPE (p = 0.051) or HR (p = 0.209), A significant protocol × time interaction was observed for BLa (p = 0.005). BLa remained lower after SIT-1 than after SIT-2, SIT-3, and SIT-4 throughout recovery and decreased from 14.47 mmol·L^-1^ immediately post-exercise to 9.96 mmol·L^-1^ at 9 min, whereas BLa remained comparatively elevated after SIT-2 and SIT-3.

**Conclusions:**

Among the four composite configurations, SIT-1 (12 × 25 m) produced the lowest early-recovery BLa, whereas SIT-2 (10 × 35 m) elicited the highest overall RPE and HR and, together with SIT-3, maintained the highest BLa. Coaches should therefore select the complete SIT set structure according to the desired acute metabolic and perceptual load and subsequent within-session recovery requirements, rather than considering repetition distance alone.

## 1 Introduction

Competitive swimming performance depends on the integration of aerobic endurance, anaerobic capacity, technical efficiency, and fatigue resistance. For middle- and long-distance swimmers, training programs must simultaneously develop physiological function while managing accumulated fatigue. Therefore, high-intensity training methods, particularly sprint interval training (SIT), have become an essential component of modern swimming training preparation (Laursen and Jenkins, 2002).

Sprint interval training is characterized by repeated maximal or near-maximal exercise bouts interspersed with recovery intervals, the duration of which may vary according to the intended training stimulus. Longer recovery intervals may be deliberately prescribed when the aim is to preserve sprint quality and maximal effort across repeated bouts. Previous studies have demonstrated that SIT can produce substantial improvements in aerobic fitness, anaerobic power, repeated-sprint ability, and metabolic efficiency while requiring considerably less training time than traditional endurance training (Tabata et al., 1996; Girard et al., 2011; Gibala et al., 2012). The physiological benefits of SIT are largely attributed to the simultaneous activation of phosphagen, glycolytic, and oxidative energy systems during repeated maximal efforts (Buchheit and Laursen, 2013). Consequently, SIT has been widely adopted by swimmers seeking to improve race performance and physiological conditioning.

In swimming, SIT is commonly prescribed using a variety of sprint distances ranging from 25 m to 100 m (Maglischo, 2003). Coaches frequently manipulate sprint distance, repetition number, and recovery duration to target specific physiological adaptations (Maglischo, 2003; Jin et al., 2026). In swimming, SIT sets are designed through the combined manipulation of repetition distance, repetition number, between-repetition recovery duration, and total sprint volume. Collectively, these variables influence the duration of each effort, the extent of phosphocreatine resynthesis and recovery between repetitions, the ability to maintain swimming velocity, and the relative contributions of phosphagen, glycolytic, and oxidative metabolism (Bogdanis et al., 1996). Consequently, each sets performed with maximal intent may elicit different metabolic, cardiovascular, and perceptual responses even when both are classified as SIT. Nevertheless, the acute physiological consequences of these composite configurations, particularly their influence on the early post-exercise recovery period, remain incompletely understood. Assessing RPE, HR, and BLa throughout early recovery may therefore provide complementary information on perceptual, cardiovascular, and metabolic strain, respectively, and help clarify how different SIT configurations shape acute internal load.

During repeated maximal exercise, both the duration of each effort and the recovery interval influence the relative contributions of the phosphagen, glycolytic, and oxidative energy systems. During the initial seconds of maximal exercise, ATP resynthesis depends substantially on phosphocreatine (PCr) degradation and anaerobic glycolysis. As maximal sprints are repeated, glycolytic contribution may decrease, whereas the relative contribution of oxidative metabolism increases (Gaitanos et al., 1993; Bogdanis et al., 1996).

The specialized evidence in swimming further indicates that different distances of maximum-intensity sprints will result in different swimming speeds, peak blood lactate concentrations, and maximum lactate accumulation rates (Mavroudi et al., 2023).These findings suggest that repetition distance alone does not determine the acute internal response; rather, its effects interact with repetition number, recovery duration, and total sprint volume. Repeated maximal efforts may additionally produce peripheral fatigue and alterations in muscle recruitment (Billaut et al., 2013), while greater anaerobic participation has been associated with slower post-exercise parasympathetic reactivation (Buchheit et al., 2007).Accordingly, the purpose of the present study was to compare RPE, HR, and BLa responses during early recovery following four composite SIT configurations differing in repetition distance, repetition number, recovery interval, and total sprint volume.

Recent research has increasingly emphasized post-exercise recovery as an important component of training monitoring because it provides valuable information regarding athletes’ physiological status and recovery capacity (Sorgente et al., 2023).Rather than relying on isolated post-exercise measurements, monitoring the dynamic changes in physiological and perceptual variables throughout the recovery process may provide additional insights into fatigue development, metabolic stress, and recovery ability. In particular, serial measurements of blood lactate concentration during early recovery may provide information regarding the magnitude and temporal evolution of post-exercise metabolic disturbance (Daanen et al., 2012). Likewise, heart rate recovery reflects autonomic nervous system function and has been widely used to assess training status and recovery capacity in athletes (Daanen et al., 2012).

Previous investigations have primarily focused on comparisons between sprint interval training and traditional endurance training or high-intensity interval training (Gibala et al., 2012; Buchheit and Laursen, 2013). Although several swimming studies have examined selected protocol components, such as repetition distance and recovery conditions, these studies have generally focused on blood lactate responses, biochemical markers, or performance outcomes rather than the serial early-recovery profiles of RPE, HR, and BLa within the same swimmers (Kostoulas et al., 2018; Kabasakalis et al., 2020; Mavroudi et al., 2023).Therefore, evidence directly comparing these responses across different composite SIT configurations remains limited. In competitive swimmers, understanding these responses is particularly important because training stress must be carefully balanced against recovery requirements (Pyne and Sharp, 2014). A recent study reported marked BLa, HR, and RPE responses during and after sprint interval swimming (Arsoniadis and Toubekis, 2024). However, it remains unclear whether commonly used SIT configurations produce different early post-exercise response patterns within the same swimmer population. Blood lactate monitoring has also been used to characterize training status and performance-related thresholds in world-ranked swimmers (Pyne et al., 2001).

A recent study reported marked BLa, HR, and RPE responses during and after sprint interval swimming (Arsoniadis and Toubekis, 2024). However, it remains unclear whether commonly used SIT configurations produce different early post-exercise response patterns within the same swimmer population. The purpose of the present study was therefore to compare RPE, HR, and BLa responses following four composite SIT configurations differing in sprint distance, repetition number, recovery interval, and total sprint volume. We hypothesized that the four configurations would elicit different acute post-exercise physiological and perceptual responses.

## 2 Methods

### 2.1 Participants

Fifteen competitive middle- and long-distance freestyle swimmers (10 males and 5 females) voluntarily participated in this study. All participants had undergone systematic swimming training for at least five years and held the National First-Class Athlete qualification in freestyle swimming under the Chinese Athlete Technical Grade System. The inclusion criteria were as follows: (1) certification as a National First-Class Athlete in freestyle swimming; (2) specialization in middle- or long-distance freestyle events; (3) participation in regular competitive training; and (4) no history of musculoskeletal injury or cardiovascular disease within the previous six months. Athletes were excluded if they experienced acute illness or injury or were unable to complete all experimental sessions.

### 2.2 Study design

The study protocol was approved by the Ethics Committee of Hunan Normal University (Approval No. 2025-560). Before participation, all athletes provided written informed consent.

**Figure 1:**
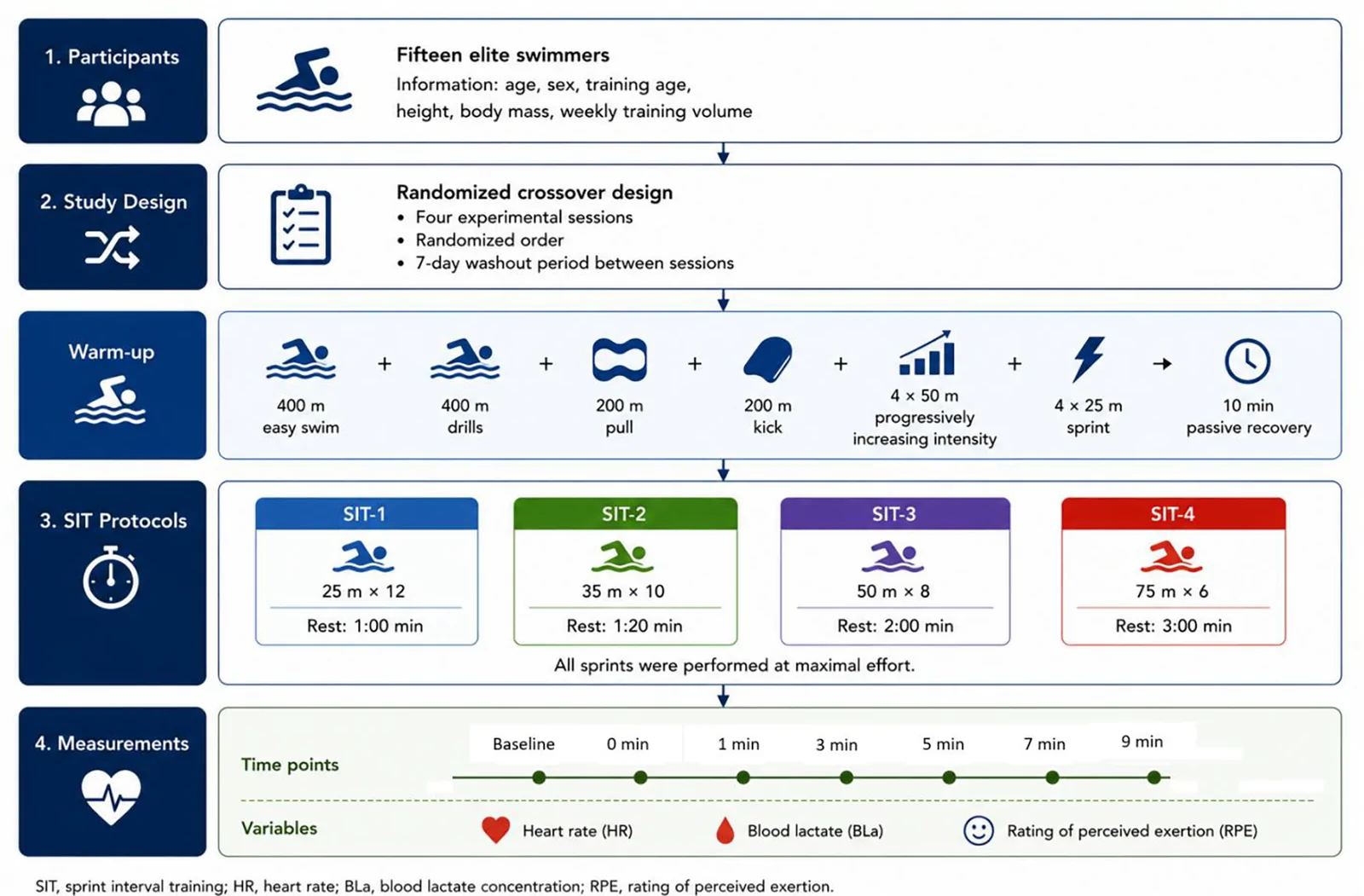
Experimental design and study protocol. Fifteen highly trained national-level swimmers participated in a randomized crossover study comprising four sprint interval training (SIT) protocols performed in a randomized order, with a 7-day washout period between sessions. Before each experimental session, participants completed a standardized warm-up consisting of 400 m of easy swimming, 400 m of swimming drills, 200 m of pull swimming, 200 m of kick swimming, 4 × 50 m of progressively increasing-intensity swimming, and 4 × 25 m of maximal sprint swimming, followed by 10 min of passive recovery. The four SIT protocols differed in sprint distance, number of repetitions, and recovery interval. Heart rate (HR), blood lactate concentration (BLa), and rating of perceived exertion (RPE) were assessed at baseline, immediately after exercise, and at 1, 3, 5, 7 and 9 min during recovery.

A randomized crossover design was employed in the present study. The study procedure is shown in **Figure 1**. Each participant completed four sprint interval training (SIT) protocols in a randomized order. The four protocols differed in sprint distance, number of repetitions, recovery interval, and total sprint distance but were designed to represent commonly used sprint interval training configurations in competitive swimming.

The experimental sessions were separated by a 7-day washout period to minimize potential carryover effects and ensure adequate recovery. All tests were conducted at the same indoor swimming pool under similar environmental conditions.

Participants were instructed to avoid strenuous exercise, caffeine, and alcohol consumption for 24 h before each experimental session. In addition, athletes were required to maintain their normal dietary habits and sleep schedules throughout the study period.

A Greenhouse–Geisser-corrected repeated-measures analysis of variance showed no statistically significant difference in TRIMP among the four protocols (F_1.77,26.55_ = 0.56, p=0.56, ηp²=0.04). This result was interpreted as an absence of evidence for a difference in TRIMP rather than as formal evidence that the protocols were equivalent. Given the limited responsiveness of HR-based load indices during short maximal exercise bouts, TRIMP was not used to establish equivalence of the four configurations.

### 2.3 Sprint interval training protocols

Prior to each experimental session, participants completed a standardized warm-up consisting of 400 m of easy swimming, 400 m of swimming drills, 200 m of pull swimming, 200 m of kick swimming, 4 × 50 m of progressively increasing-intensity swimming, and 4 × 25 m of maximal sprint swimming to re-familiarize themselves with maximal-effort sprinting and enhance neuromuscular readiness. After a 10-min passive recovery period, participants completed one of the SIT protocols shown in **Table 1**. Each participant completed four SIT configurations in randomized order: 12 × 25 m with 1-min recovery, 10 × 35 m with 80-s recovery, 8 × 50 m with 2-min recovery, and 6 × 75 m with 3-min recovery. All sprint repetitions were performed at maximal effort. Verbal encouragement was provided throughout the protocols to ensure maximal motivation and performance. All experimental sessions were performed at the same time of day in a 50-m indoor swimming pool. All trials were completed using the freestyle stroke, with each sprint initiated from an in-water push-off. The water temperature was maintained at 25–26 °C throughout all experimental sessions.

**Table 1:** Sprint interval training (SIT) protocols.

Protocol	Sprint distance (m)	Number of repetitions	Recovery interval	Total sprint distance (m)
SIT-1	25	12	1 min	300
SIT-2	35	10	80 s	350
SIT-3	50	8	2 min	400
SIT-4	75	6	3 min	450

### 2.4 Outcome measures

#### 2.4.1 Rating of perceived exertion

Perceived exertion was assessed using the Borg 6–20 Rating of Perceived Exertion (RPE) scale. Participants were familiarized with the scale before the experiment. RPE was recorded at baseline (Pre), immediately after exercise (Post), and at 1, 3, 5, 7, and 9 min during recovery. Baseline measurements were obtained after the standardized warm-up and subsequent 10-min passive recovery, immediately before the SIT protocol.

#### 2.4.2 Heart rate

Heart rate (HR) was continuously monitored throughout each experimental session using a waterproof heart rate monitor (SR834, Solver Sports Technology Co., Ltd., Shenzhen, China). Resting HR was recorded before exercise. Post-exercise HR values were extracted immediately after exercise and at 1, 3, 5, 7, and 9 min during recovery.

#### 2.4.3 Blood lactate concentration

Capillary blood samples (approximately 10 μL) were collected from the fingertip to determine blood lactate concentration (BLa). Samples were obtained at baseline (Pre), immediately after exercise, and at 1, 3, 5, 7, and 9 min following exercise. Blood lactate concentration was analyzed using a portable lactate analyzer (Catalog No. 5214-0051-2200, EKF-diagnostic GmbH, Barleben, Germany). The analyzer was calibrated before each testing session according to the manufacturer’s instructions.

### 2.5 Statistical analysis

All statistical analyses were performed using R software (Version 4.3.0; R Foundation for Statistical Computing, Vienna, Austria). Continuous variables are presented as mean ± standard deviation (SD), unless otherwise stated. Before analysis, the dataset was examined for duplicate observations, missing values, invalid protocol or time labels, and physiologically implausible values. Missing values were not imputed.

Linear mixed-effects models were used to examine post-exercise RPE, HR, and BLa. The primary recovery analysis included the six time points consistently available for all four protocols: Post and 1, 3, 5, 7, and 9 min. Time was treated as a categorical variable to avoid imposing a linear recovery trajectory. Protocol, time, and the protocol × time interaction were included as fixed effects. The session-specific Pre value was grand-mean centered and entered as a covariate. Random intercepts were initially specified for participant and experimental session, with session defined as each participant-by-protocol combination, to account for correlations among repeated observations from the same participant and within the same experimental session. If this random-effects structure produced a singular fit, a simplified model containing only a participant random intercept was prespecified. The full participant and session random-intercept structure was retained for all three primary outcomes.

Models were estimated using restricted maximum likelihood. For the RPE and raw-scale BLa models, fixed effects were assessed using Type III F tests with Kenward–Roger-adjusted denominator degrees of freedom. The Satterthwaite approximation was used only when the Kenward–Roger procedure could not be computed.

When the protocol × time interaction was statistically significant, between-protocol comparisons were performed separately at each recovery time point using estimated marginal means, with Bonferroni adjustment for the six pairwise protocol comparisons. When the interaction was not statistically significant, the overall protocol effect averaged across the six recovery time points was examined using Bonferroni-adjusted pairwise comparisons. Adjusted mean differences, 95% confidence intervals (CIs), and multiplicity-adjusted p values were reported.

Model adequacy was evaluated by examining convergence, singularity, residual normality, heteroscedasticity, residual-versus-fitted plots, and potentially influential observations. Observations were not excluded solely on the basis of statistical diagnostic flags.

Because the initial HR model showed evidence of nonconstant residual variance, an additional model was fitted using the nlme package, allowing the residual variance to differ across recovery time points through a varIdent variance structure. The time-specific heteroscedastic model was compared with an otherwise identical homoscedastic model using the likelihood-ratio test and Akaike information criterion (AIC). Because the heteroscedastic model provided a significantly better fit, its estimates were used for the final HR inference.

Because the residuals of the raw-scale BLa model deviated from normality, a natural-log-transformed BLa model was performed as a sensitivity analysis. The corresponding session-specific Pre BLa value was also natural-log transformed and centered. Protocol, time, and protocol × time conclusions from the raw-scale and log-transformed models were compared. Geometric mean ratios and their 95% CIs were used for time-specific comparisons in the log-transformed model. Raw-scale estimates were retained as the primary results to facilitate clinical and practical interpretation. All tests were two-sided, and statistical significance was set at p<0.05. Exact p values are reported except when p<0.001.

## 3 Results

### 3.1 Participant characteristics

The characteristics of the participants are presented in **Table 2**. Fifteen national-level competitive middle- and long-distance freestyle swimmers (10 males and 5 females) participated in the study. According to the Participant Classification Framework, the participants were classified as Tier 3 (Highly Trained/National Level) (McKay et al., 2022), because they represented their universities in national-level competitions. All participants also held the National First-Class Athlete qualification in freestyle swimming under the Chinese Athlete Technical Grade System and had completed at least five years of systematic swimming training. Their mean age, height, body mass, body mass index, and skeletal muscle mass were 19.50 ± 1.63 years, 177.38 ± 6.87 cm, 71.37 ± 8.31 kg, 22.67 ± 1.67 kg·m^-2^, and 33.72 ± 6.04 kg, respectively. To provide an objective indication of competitive performance, personal-best freestyle times achieved within the 12 months preceding the study were also recorded. The male swimmers achieved mean personal-best times of 25.63 ± 0.32 s, 54.99 ± 0.46 s, and 121.30 ± 0.70 s in the 50-m, 100-m, and 200-m freestyle events, respectively. The corresponding times for the female swimmers were 26.24 ± 0.39 s, 56.48 ± 0.47 s, and 125.60 ± 0.74 s, respectively. Together, these classification and performance data characterize the participants as highly trained national-level university swimmers. Accordingly, the present findings are most applicable to highly trained national-level university swimmers and should be generalized cautiously to recreational swimmers, international-level athletes, or swimmers specializing in other competitive distances.

**Table 2:** Characteristics of the participants (n = 15).

Variable	Mean ± SD
Age (years)	19.50 ± 1.63
Height (cm)	177.38 ± 6.87
Body mass (kg)	71.37 ± 8.31
Body mass index (kg·m^−2^)	22.67 ± 1.67
Skeletal muscle mass (kg)	33.72 ± 6.04
Waist-to-hip ratio	0.84 ± 0.03
Training Volume (km/week)	48.6 ± 6.22

### 3.2 Rating of perceived exertion

**Figure 2:**
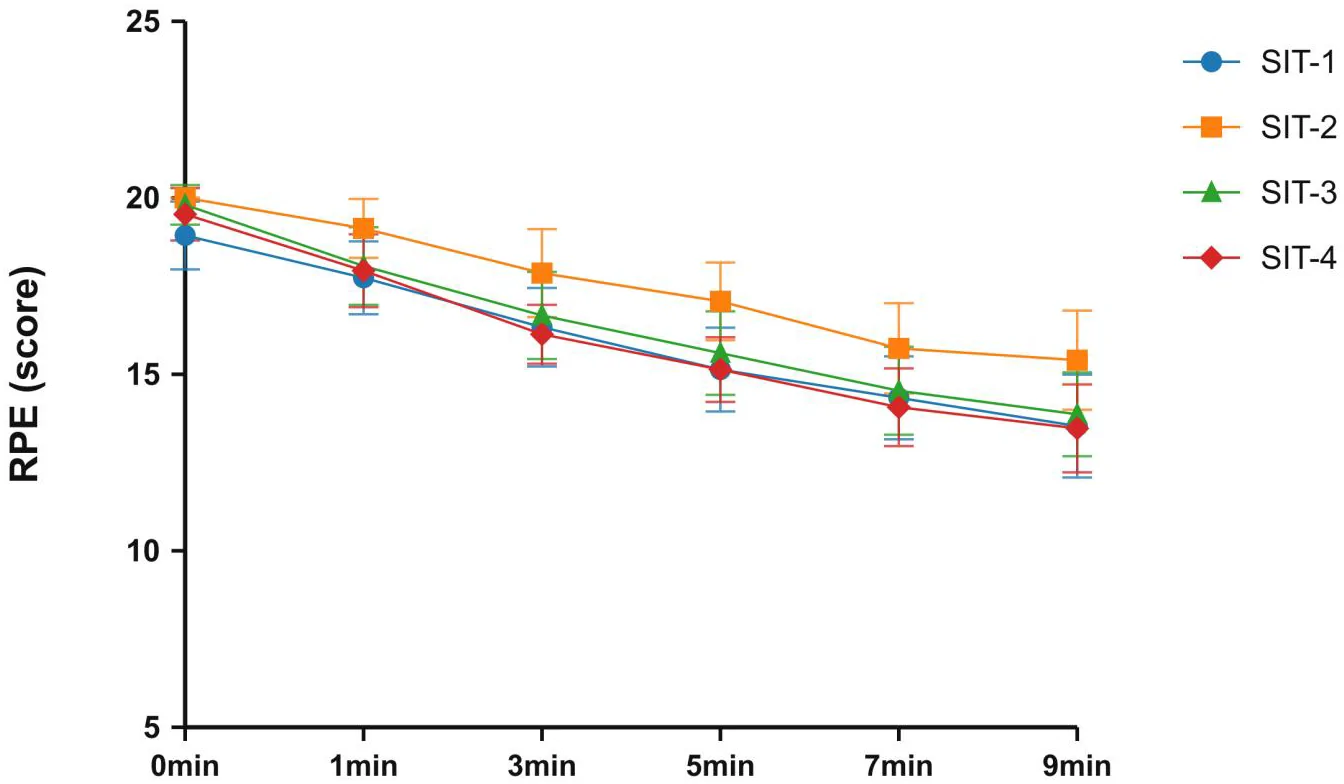
Changes in rating of perceived exertion (RPE) during post-exercise recovery following four sprint interval training (SIT) protocols. RPE was assessed immediately after exercise (0 min) and at 1, 3, 5, 7, and 9 min of recovery following each protocol (SIT-1: 12 × 25 m; SIT-2: 10 × 35 m; SIT-3: 8 × 50 m; SIT-4: 6 × 75 m). Symbols represent means, and vertical error bars represent ±1 SD.

Post-exercise RPE responses are presented in **Figure 2**. The linear mixed-effects model revealed significant main effects of protocol (F_3,41.49_ = 26.84, p<0.001) and recovery time (F_5,280_ = 434.58, p<0.001) on post-exercise RPE. The protocol × time interaction did not reach statistical significance (F_15,280_ = 1.70, p=0.051). The session-specific Pre RPE covariate was not significant (F_1,54.52_ = 0.44, p=0.508).

Bonferroni-adjusted comparisons averaged across recovery time points showed that RPE was higher following SIT-2 (10 × 35 m) than following SIT-1 (12 × 25 m; adjusted mean difference = 1.54 units, p<0.001), SIT-3 (8 ×50 m; adjusted mean difference = 1.09 units, p<0.001), and SIT-4 (6 × 75 m; adjusted mean difference = 1.48 units, p<0.001). No statistically significant differences were observed among SIT-1, SIT-3, and SIT-4 (all adjusted p\geq0.173).

Immediately after exercise, the adjusted RPE values were 18.92 for SIT-1, 19.99 for SIT-2, 19.81 for SIT-3, and 19.54 for SIT-4. At 9 min, the corresponding values were 13.52, 15.39, 13.88, and 13.47, respectively. Therefore, SIT-2, rather than the 25-m protocol, elicited the greatest overall post-exercise perceptual response.

### 3.3 Heart rate

**Figure 3:**
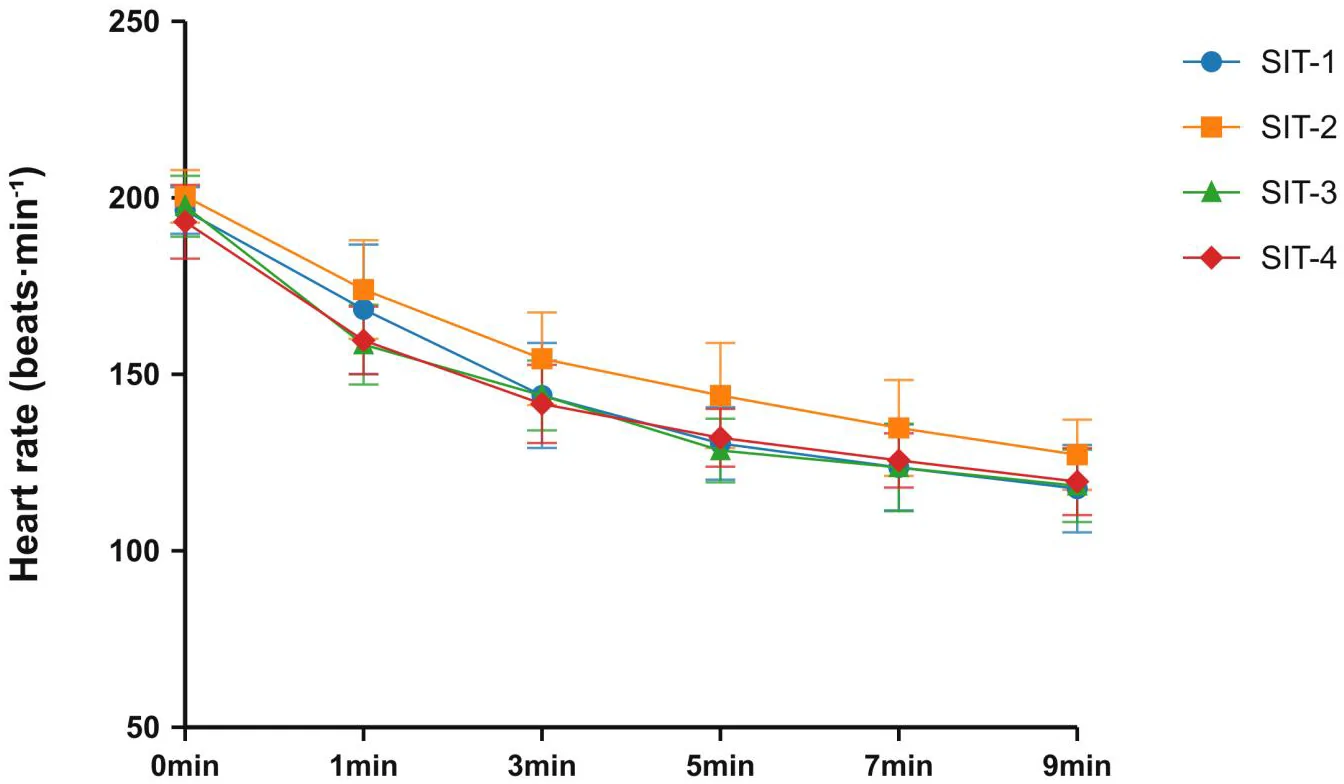
Changes in heart rate (HR) during post-exercise recovery following four sprint interval training (SIT) protocols. HR was assessed immediately after exercise (0 min) and at 1, 3, 5, 7, and 9 min of recovery following each protocol (SIT-1: 12 × 25 m; SIT-2: 10 × 35 m; SIT-3: 8 × 50 m; SIT-4: 6 × 75 m). Symbols represent means, and vertical error bars represent ±1 SD.

Post-exercise heart rate responses are presented in **Figure 3**, and the corresponding linear mixed-effects model results are shown in **Table 3**. Because the initial HR model showed nonconstant residual variance, an otherwise identical model allowing residual variance to differ across recovery time points was fitted using a varIdent structure and compared with the homoscedastic model using a likelihood-ratio test. The time-specific heteroscedastic model was selected for final HR inference because it accommodated the observed residual variance pattern and provided acceptable residual diagnostics.

**Table 3:** Results of the linear mixed-effects models examining the effects of protocol, time, and protocol × time interaction on post-exercise physiological and fatigue responses.

Outcome	Effect	F	df	p
RPE	Protocol	26.84	3,41.49	<0.001
	Time	434.58	5,280	<0.001
	Protocol × Time	1.70	15,280	0.051
Heart rate (bpm)	Protocol	7.35	3,41	<0.001
	Time	532.08	5,280	<0.001
	Protocol × Time	1.29	15,280	0.209
Blood lactate (mmol·L^−1^)	Protocol	77.91	3,41.05	<0.001
	Time	62.89	5,280	<0.001
	Protocol × Time	2.28	15,280	0.005

Significant main effects of protocol (F_3,41_ = 7.35, p<0.001) and recovery time (F_5,280_ = 532.08, p<0.001) were observed for post-exercise HR. The protocol × time interaction was not significant (F_15,280_ = 1.29, p=0.209), and the session-specific Pre HR covariate was not significant (F_1,41_ = 0.07, p=0.795). Thus, HR decreased substantially during recovery in all four protocols, with no evidence that the overall recovery trajectory differed between protocols.

Averaged across recovery time points, HR was higher following SIT-2 than following SIT-1 by 9.03 beats·min^-1^ (95% CI 1.67–16.39, adjusted p=0.009), SIT-3 by 10.75 beats·min^-1^ (95% CI 3.40–18.11, adjusted p=0.001), and SIT-4 by 10.44 beats·min^-1^ (95% CI 3.03–17.86, adjusted p=0.002). No statistically significant differences were observed among SIT-1, SIT-3, and SIT-4 (all adjusted p=1.000).

Adjusted HR values ranged from 193.26 to 200.37 beats·min^-1^ immediately after exercise and from 117.61 to 127.17 beats·min^-1^ at 9 min.

### 3.4 Blood lactate concentration

**Figure 4:**
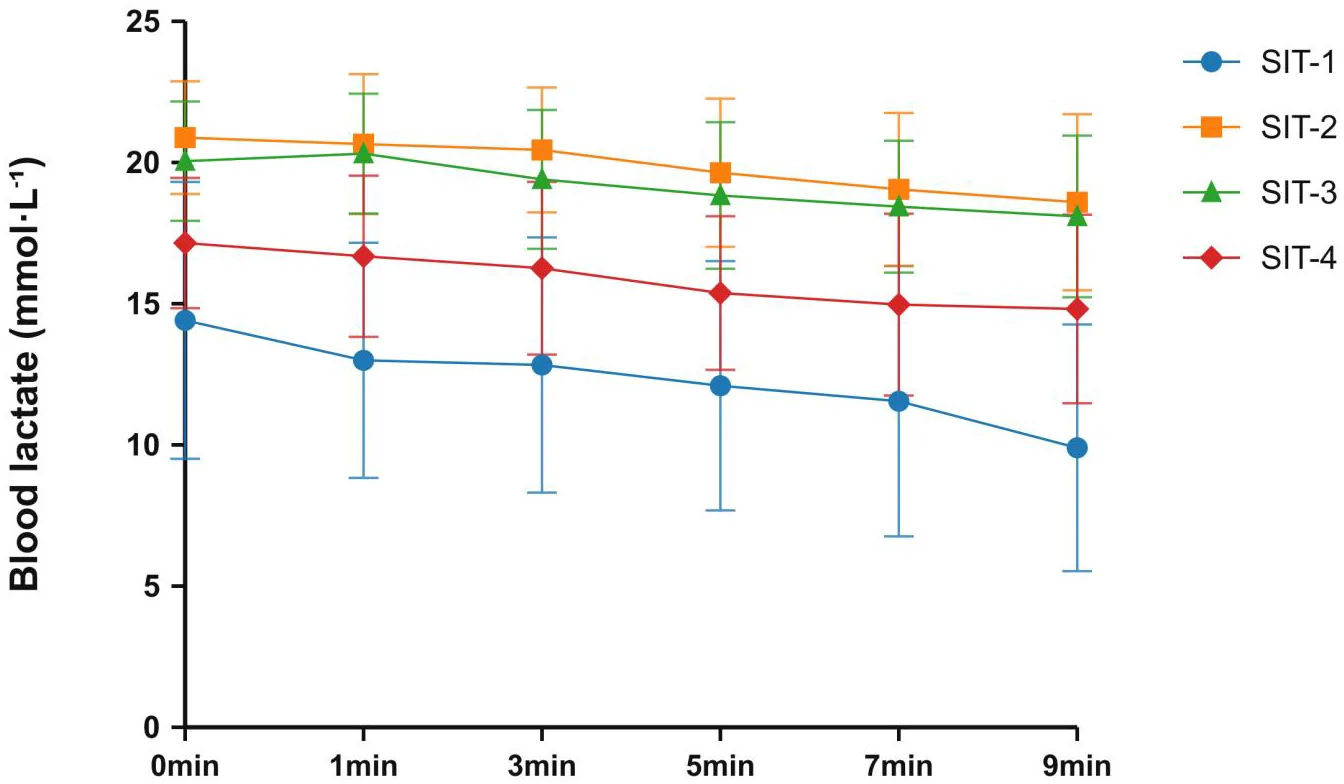
Changes in blood lactate concentration (BLa) during post-exercise recovery following four sprint interval training (SIT) protocols. BLa was assessed immediately after exercise (0 min) and at 1, 3, 5, 7, and 9 min of recovery following each protocol (SIT-1: 12 × 25 m; SIT-2: 10 × 35 m; SIT-3: 8 × 50 m; SIT-4: 6 × 75 m). Symbols represent means, and vertical error bars represent ±1 SD.

Post-exercise BLa responses are presented in **Figure 4**. The raw-scale BLa model demonstrated significant main effects of protocol (F_3,41.05_ = 77.91, p<0.001) and recovery time (F_5,280_ = 62.89, p<0.001), together with a significant protocol × time interaction (F_15,280_ = 2.28, p=0.005). The session-specific Pre BLa covariate was not significant (F_1,44.46_ = 2.57, p=0.116). Because the interaction was significant, between-protocol differences were examined separately at each recovery time point.

SIT-1 produced lower adjusted BLa concentrations than SIT-2, SIT-3, and SIT-4 at every recovery time point from Post to 9 min (all Bonferroni-adjusted p ≤ 0.001). SIT-2 and SIT-3 did not differ at any recovery time point (all adjusted p≥0.998). In the raw-scale model, both SIT-2 and SIT-3 produced higher BLa concentrations than SIT-4 at all recovery time points (all adjusted p<0.001).

Immediately after exercise, adjusted BLa concentrations were 14.47 mmol·L^-1^ (95% CI 12.74–16.21) following SIT-1, 20.82 mmol·L^-1^ (95% CI 19.08–22.55) following SIT-2, 20.11 mmol·L^-1^ (95% CI 18.38–21.85) following SIT-3, and 17.08 mmol·L^-1^ (95% CI 15.35–18.82) following SIT-4. At 9 min, the corresponding concentrations were 9.96 mmol·L^-1^ (95% CI 8.23–11.70), 18.53 mmol·L^-1^ (95% CI 16.80–20.26), 18.16 mmol·L^-1^ (95% CI 16.42–19.89), and 14.75 mmol·L^-1^ (95% CI 13.01–16.48), respectively.

The log-transformed sensitivity model retained significant effects of protocol (F_3,41.08_ = 39.25, p<0.001), recovery time (F_5,280_ = 50.12, p<0.001), and protocol × time interaction (F_15,280_ = 5.33, p<0.001), whereas the transformed Pre covariate remained nonsignificant (F_1,47.81_ = 2.68, p=0.108). Thus, all omnibus conclusions were consistent between the raw-scale and log-transformed models.

Most time-specific comparisons were also unchanged. However, the immediately post-exercise difference between SIT-3 and SIT-4 did not remain statistically significant after log transformation and Bonferroni adjustment (geometric mean ratio = 1.19, 95% CI 1.00–1.41, adjusted p=0.053). This comparison was therefore interpreted cautiously. From 1 to 9 min, BLa remained significantly higher following SIT-3 than following SIT-4 (all adjusted p ≤ 0.016).

## 4 Discussion

The present study compared early post-exercise physiological and perceptual responses following four sprint interval training (SIT) configurations in middle- and long-distance swimmers. After adjustment for session-specific baseline values, the clearest configuration-dependent temporal difference was observed for blood lactate concentration (BLa), which demonstrated a significant protocol × time interaction. In contrast, RPE and HR showed significant protocol and time effects, but no statistically significant protocol × time interaction was detected for either outcome. SIT-2 elicited the highest overall RPE and HR responses across the post-exercise period, whereas SIT-1 consistently produced the lowest BLa concentrations. Importantly, these findings reflect responses to four composite SIT configurations rather than the isolated effect of sprint distance, because sprint distance, repetition number, recovery interval, and total sprint volume varied simultaneously among protocols.

### 4.1 Perceptual and cardiovascular recovery

Post-exercise RPE differed significantly among the four SIT configurations, with SIT-2 eliciting the highest overall perceived exertion across the six recovery time points. Although the protocol × time interaction did not reach statistical significance (p = 0.051), the significant protocol effect suggests that SIT-2 imposed a greater overall perceptual demand during early recovery. This perceptual response should be interpreted alongside the HR and BLa findings, which provide complementary information regarding the cardiovascular and metabolic demands of the four configurations. Previous studies have shown that perceptual fatigue is strongly associated with metabolic perturbations during high-intensity exercise, including lactate accumulation, hydrogen ion concentration, and alterations in muscle homeostasis (Borg, 1998). Marcora et al. further proposed that perceived exertion reflects the integration of multiple central and peripheral signals rather than exercise duration alone (Marcora, 2009). Similarly, Pageaux suggested that perceived effort is influenced by both physiological stress and psychological regulation during exercise (Pageaux, 2016). Therefore, the greater perceptual fatigue observed following SIT-2 may be attributable to the greater metabolic stress imposed by this protocol.

Post-exercise HR showed a corresponding protocol effect, with SIT-2 eliciting the highest overall HR response. The repeated performance of ten maximal 35-m efforts with 80-s recovery intervals may have maintained cardiovascular activation throughout the set, resulting in a higher post-exercise HR. Nevertheless, HR declined markedly and followed a broadly similar recovery pattern after all four configurations. The absence of a protocol × time interaction suggests that, after exercise cessation, parasympathetic reactivation and sympathetic withdrawal may have progressed at comparable rates across configurations, despite differences in the overall magnitude of the HR response (Buchheit and Gindre, 2006; Stanley et al., 2013; Michael et al., 2017).However, because cardiac autonomic activity was not directly assessed using HR variability or related measures, this explanation remains inferential. Together, the RPE and HR findings suggest that the SIT configurations primarily affected the magnitude of the perceptual and cardiovascular responses rather than their early recovery trajectories.

The significant protocol effect indicates that the overall post-exercise HR level differed among configurations, whereas the nonsignificant interaction indicates only that configuration-dependent differences in the temporal HR pattern were not detected. This finding may reflect the superior autonomic regulation and recovery capacity of highly trained national-level swimmers who are accustomed to repeated high-intensity training stimuli (Stanley et al., 2013).

### 4.2 Early post-exercise blood lactate responses

The clearest configuration-dependent temporal response was observed for BLa, which showed a significant protocol × time interaction in the baseline-adjusted model. This finding was robust to the sensitivity analysis using log-transformed BLa, in which the protocol × time interaction also remained statistically significant. However, the immediately post-exercise comparison between SIT-3 and SIT-4 became borderline after log transformation and Bonferroni adjustment, indicating that this specific contrast should be interpreted cautiously.

SIT-2 and SIT-3 produced the highest BLa responses, although the underlying physiological demands may have differed between these configurations. Repeated-sprint studies have shown that PCr availability, glycolytic ATP production, and oxidative energy contribution change across successive maximal efforts and are influenced by the duration of the intervening recovery (Gaitanos et al., 1993; Bogdanis et al., 1995; Bogdanis et al., 1996). In SIT-2, the combination of ten 35-m maximal efforts and 80-s recovery intervals may therefore have resulted in repeated glycolytic activation before complete metabolic recovery. In SIT-3, the longer 50m repetitions and greater total sprint volume may have increased the glycolytic demand of each effort despite the longer 2-min recovery interval. This interpretation is supported by swimming-specific evidence showing that maximal 25m, 35m, and 50m efforts produce different peak BLa concentrations and maximal lactate accumulation rates, with the highest peak BLa observed after 50 m (Mavroudi et al., 2023).These opposing combinations of repetition duration, repetition number, recovery duration, and total volume may explain why SIT-2 and SIT-3 produced similarly high circulating BLa concentrations (Gaitanos et al., 1993; Bogdanis et al., 1995; Bogdanis et al., 1996; Toubekis and Tokmakidis, 2013).

In contrast, SIT-1 consistently produced the lowest BLa response. The shorter 25-m repetitions would be expected to rely relatively more on phosphagen availability during the initial phase of each effort and to produce less total lactate accumulation per repetition (Gaitanos et al., 1993; Mavroudi et al., 2023) The 1-min recovery interval may also have permitted partial PCr resynthesis between these short efforts, as PCr recovery occurs rapidly during the first minutes following maximal exercise and is closely associated with the restoration of sprint power (Bogdanis et al., 1995). Combined with the lowest total sprint volume (300 m), these factors provide a plausible explanation for the lower BLa observed following SIT-1. However, because PCr resynthesis and energy-system contributions were not directly measured, this interpretation should remain cautious.

The longest repetition distance in SIT-4 did not produce the highest BLa response. Although SIT-4 had the greatest total sprint volume, it included only six repetitions and the longest recovery interval (3 min). The longer recovery may have permitted additional PCr resynthesis and greater metabolic recovery before the subsequent repetition (Bogdanis et al., 1995). Swimming-specific research has also shown that the duration and type of recovery interval can influence repeated-sprint performance and post-exercise BLa responses (Kostoulas et al., 2018). In addition, a reduction in swimming velocity during the longer 75-m efforts could have been accompanied by a greater relative oxidative contribution and a reduced glycolytic rate during the later stages of each repetition (Gaitanos et al., 1993; Bogdanis et al., 1996). Because repetition times, velocity, pacing, and performance decrement were not recorded, these explanations remain speculative. More broadly, previous swimming research has shown that differences in repetition distance do not necessarily produce corresponding differences in post-exercise biochemical markers when other set characteristics are controlled (Kabasakalis et al., 2020). This further supports interpreting the present findings at the level of the complete SIT configuration rather than repetition distance alone.

Collectively, the significant protocol × time interaction for BLa likely reflects configuration-specific differences in the balance between lactate appearance, redistribution, oxidation, and removal during early recovery (Pelayo et al., 1996; Vescovi et al., 2011) BLa therefore appeared to be more sensitive than RPE or HR to differences among the four composite SIT configurations. However, the findings cannot identify the independent contribution of repetition distance, repetition number, recovery interval, or total sprint volume.

### 4.3 Practical applications

The present findings may have practical implications for the organization of SIT within individual swimming training sessions. SIT-1 produced the lowest early post-exercise BLa concentrations, consistent with a lower circulating lactate response, although this should not be interpreted as evidence of a lower total internal training load.SIT-2 elicited the highest overall RPE and HR responses and was accompanied by high BLa concentrations, indicating that coaches may consider allowing sufficient recovery before subsequent technically demanding work within the same session. Because SIT-2 and SIT-3 both produced high BLa responses, subsequent training content requiring precise technical execution may need to be scheduled with consideration of the acute physiological disturbance produced by these sets. Accordingly, coaches should consider the complete structure of a SIT set—including repetition distance, number of repetitions, recovery interval, and total volume—rather than using repetition distance alone to anticipate the acute post-exercise response. These interpretations are limited to acute session organization and should not be extrapolated to long-term training adaptations.

### 4.4 Limitations

Several limitations of the present study should be acknowledged. First, the four SIT configurations differed simultaneously in repetition distance, number of repetitions, recovery interval, and total sprint volume. Consequently, the independent contribution of each component cannot be isolated, and the present findings should be interpreted as responses to composite SIT configurations rather than as effects of sprint distance alone. Second, the sample size was relatively small, which limits the precision of effect estimates, particularly for protocol × time interactions. In addition, the inclusion of both male and female swimmers did not permit adequately powered sex-specific analyses. Third, sprint times, swimming velocity, performance decrement, pacing strategy, stroke mechanics, PCr resynthesis, energy-system contributions, and neuromuscular fatigue were not quantified. Therefore, the proposed physiological explanations remain mechanistic interpretations rather than directly demonstrated causal mechanisms. Therefore, the mechanisms underlying the observed configuration-specific physiological responses cannot be determined from the present data. Fourth, the primary post-exercise observation period extended only to 9 min. The present findings therefore characterize early post-exercise responses rather than complete physiological or metabolic recovery. Fifth, although protocol order was randomized and sessions were separated by a 7-day washout period, residual period or carryover effects cannot be completely excluded. Finally, the study examined acute responses only. Longitudinal intervention studies are required to determine whether the acute differences observed among configurations translate into different chronic training adaptations.

## 5 Conclusions

Four composite SIT configurations elicited different acute post-exercise physiological and perceptual responses in middle- and long-distance swimmers. After adjustment for session-specific baseline values, BLa demonstrated a significant protocol × time interaction, whereas no statistically significant interaction was detected for RPE or HR. SIT-1 consistently produced the lowest BLa concentrations, whereas SIT-2 elicited the highest overall RPE and HR responses. Because sprint distance, repetition number, recovery interval, and total sprint volume varied simultaneously, these findings should be interpreted as configuration-specific responses rather than isolated effects of sprint distance.

## Data Availability

The raw data supporting the conclusions of this article will be made available by the authors, without undue reservation.
